# Microbiome dysbiosis in spinal pathology: Mechanisms, evidence, and research limitations

**DOI:** 10.1016/j.bas.2025.104272

**Published:** 2025-05-02

**Authors:** Muaz Rashid, Hugo Serra Pereira, Ahmad Alissa, Salman Keraidi, Nicolas Wipf, Aubrie M. Sowa, Jake M. McDonnell, Stacey Darwish, Joseph S. Butler

**Affiliations:** a School of Medicine, University College Dublin, Dublin, Ireland; b National Spinal Injuries Unit, Mater Misericordiae University Hospital, Dublin, Ireland; c School of Medicine, Trinity College Dublin, Dublin, Ireland; d Trinity Centre for Biomedical Engineering, Trinity Biomedical Sciences Institute, Trinity College Dublin, The University of Dublin, Dublin, Ireland; e Department of Orthopaedics, St. Vincent's University Hospital, Dublin, Ireland

**Keywords:** Spine, Ankylosing spondylitis, Spondyloarthropathy, Gut microbiome, HLA-B27, Autoimmunity

## Abstract

**Introduction:**

The microbiome's relevance has become increasingly discussed amid the rising prevalence of chronic illnesses. Microbiome research to date focuses predominantly on its relationship with the GI tract while largely ignoring any impact on the rest of the body. This narrative review aims to lay a foundation of knowledge to fill this gap in the literature and discuss other microbiomes within the human body and their relation to spinal health.

**Research question:**

What is the relationship between the human microbiome and spinal pathologies?

**Materials and methods:**

A narrative review of all available literature (written or translated to English) was performed using PubMed, MEDLINE, and Google Scholar using relevant search terms including: “microbiome”, “spine”, “spinal pathology”, “ankylosing spondylitis”, and “seronegative arthropathies”.

**Results:**

This review found that with dysbiosis, specific bacterial such as *Bacteroidaceae* and *Rikenellaceae* proliferate, altering the cytokine microenvironment and subsequently increasing gut wall permeability. This immune overactivation and improper cell function results in an increased susceptibility to autoimmunity; specifically ankylosing spondylitis and seronegative arthropathies. This review also highlights the significant gaps in the available literature.

**Discussion and conclusion:**

This review aims to equip clinicians with an understanding of how the collection of microbiomes in the human body have specific implications for spinal health. By building on the current literature and integrating this knowledge into practice, more patient-specific practices in the treatment of spinal pathologies can be implemented, ultimately improving and optimizing patient care in a field in which the microbiome is not currently at the forefront of pathology.

## Highlights

• There is a strong connection between spondyloarthropathies and gut inflammation (Speca and Dubuquoy, 2017).
• Human intervertebral discs have a distinct microbiome that is altered in disease (Rajasekaran et al., 2020).
• Improper dendritic cell processing might cause SpA (Berthelot and Claudepierre, 2016; Krajewska-Włodarczyk et al., 2017).
• In spondyloarthropathies, preliminary trials show that probiotics may reduce disease (Cammarota et al., 2014).

## 1 Introduction

### 1.1 Background and importance

The prevalence of chronic disease has seen a significant increase globally over the past decades. This burden affects nearly all body systems, and the spine is no exception; with literature demonstrating a substantial rise in spine-related pathology over the last 15 years (Kobayashi et al., 2022; Grotle et al., 2019). There is debate regarding the cause of this rise, with a growing body of research focusing on lifestyle factors and the interactions between the environment and health. Chief among these factors is the microbiome, the vast collection of microorganisms that inhabit the gastrointestinal tract but also joint spaces, skin, lungs, and various other organ systems.

The human microbiome has been demonstrated to influence a range of physiological processes that extend beyond the gastrointestinal tract, such as metabolism, immunity, and behaviour (Cresci and Bawden, 2015; Cryan et al., 2019). The gut microbiome has been shown to regulate host immunity, aid in maintaining homeostasis, and prevent immune overactivation. Its dysfunction can contribute to autoimmune and inflammatory diseases (Miyauchi et al., 2023; Shaheen et al., 2022).

### 1.2 Objective and scope

The objective of this review is to use the available literature to discuss the impact of the gut microbiome, specifically regarding ankylosing spondylitis (AS) and spondyloarthropathies (SpA). AS and SPA were specifically chosen due to their established ties with both gut dysbiosis and spine inflammation, especially through genetic associations with HLA-B27. Other spine conditions, such as degenerative disc disease and malignancy, were excluded due to the literature surrounding these topics lacking consistent microbiome-related pathophysiology. The narrow focus of this review allows for a more in-depth exploration of the microbiome and immune-associated mechanisms surrounding AS and SPA.

Furthermore, the review will address gaps in the literature and highlight gaps in research and systematic problems in the methodology of papers surrounding this topic.

## 2 Materials and methods

This narrative review aims to synthesize all available literature to (1) lay a foundation of knowledge on the microbiome, it's role in immune regulation, and its impact on bone health; and (2) discuss other microbiomes within the human body and their relation to spinal health and the development of spinal pathologies rooted in abnormal immune regulation such as ankylosing spondylitis and other seronegative arthropathies.

A literature search in PubMed, MEDLINE, and Google Scholar was conducted where all available articles were reviewed. Relevant search terms used included “microbiome”, “spine”, “spinal pathology”, “spine disease”, “autoimmune”, “autoimmunity”, “ankylosing spondylitis”, and “seronegative arthropathies”.

From this search, studies were included if they examined the role of the gut microbiome in spinal, autoimmune, or skeletal conditions. Cell, animal, and human studies were all included for consideration. Exclusion criteria were articles not written in English or the focus was not on inflammatory spine pathology. A narrative rather than systematic structure was used due to the exploratory nature of the topic.

## 3 Theory: Significance of the microbiome

### 3.1 The role of the microbiome in immune regulation

The microbiome plays a role in modulating the host immune response and maintaining gut barrier integrity, thereby influencing various aspects of health, including bone health. Dysbiosis is defined as any imbalance in the makeup of the microbiome, it can be beneficial or harmful, and may follow events such as antibiotic use or any other factor that may alter microbial composition (Shaheen et al., 2022; Annunziato et al., 2015). This imbalance can disrupt the normal immune processes and lead to immune dysregulation. As a result, a proinflammatory state can develop, increasing the body's susceptibility to autoreactivity and the development of autoimmune conditions (Shaheen et al., 2022; Annunziato et al., 2015; Gracey et al., 2019).

The immune system can be separated into three branches, which are directed against different pathogens (Annunziato et al., 2015; Gracey et al., 2019). These branches are composed of unique innate and adaptive immune cells and branch-specific effector molecules. Type 1 immunity protects against intracellular bacteria and viruses, while Type 2 immunity protects against helminths and exogenous toxins. Type 3 immunity is the branch responsible for epithelial and mucosal defence, being specialized to target extracellular bacteria and fungi. It is the Type 3 immune branch, characterized by TH17 CD4^+^ T cells, which produce Interleukin-17 (IL-17) and IL-22 that is likely responsible for the inflammatory and autoimmune changes seen in dysbiosis (Annunziato et al., 2015; Gracey et al., 2019).

### 3.2 The microbiome and signalling molecules

Despite being somewhat contained and limited to select sites, the commensal microbiome exerts its influence on host physiology through the regulation of cytokines. In dysbiotic individuals, decreased amounts of anti-inflammatory IL-10 and increased quantities of proinflammatory TNF-α and IL-17 have been measured, suggesting a possible link between microbiome disruption and proinflammatory states. This imbalance in the cytokine profile contributes to the heightened state of inflammation characteristic of the early manifestations of autoimmune conditions like AS and SpA (Fert et al., 2014; Taurog et al., 2016).

Short-chain fatty acids (SCFA), produced by intestinal flora, are hypothesized to promote immune suppression as part of the body's repertoire of anti-inflammatory signalling molecules. They are thought to promote the growth and differentiation of regulatory immune cells (Gracey et al., 2019; Speca and Dubuquoy, 2017; Asquith et al., 2017). Butyrate in particular provides a protective function to gut barrier integrity and prevents bacterial translocation into systemic circulation (Gracey et al., 2019; Speca and Dubuquoy, 2017; Asquith et al., 2017). Rodent studies have shown propionate supplementation reduces colonic inflammation, and conversely, depletion of the microbiome with oral vancomycin has resulted in a severe reduction in the levels of colonic SCFA (Asquith et al., 2017).

Certain proinflammatory cytokines such as IL-17, IL-23, and TNF-α have a hand in the development of bone and gut inflammatory disease, leading to an increased level of attention being directed to this relationship and what the role is of immune cells in the development of simultaneous gut and musculoskeletal pathology (Hand et al., 2016). Innate-like immune cells such as invariant natural killer T (iNKT) cells and mucosal-associated T (MAIT) cells are first-line responders and have a massive proinflammatory response upon stimulation by extracellular microbes. These cells have been seen to have a shared role in the development of IBD, rheumatoid arthritis (RA) and SpA. Specifically, iNKT cells have been well-documented to play a role in IBD pathogenesis, however, their precise role in the development of SpA remains unclear. MAIT cells have been implicated in gut and joint disease, with an increased number of cells being observed in pathologic gut and synovial fluid in IBD and RA/SpA respectively (Pedersen et al., 2014; Horta-Baas et al., 2017; Rudwaleit et al., 2011; Lanier, 2013; Moustafa et al., 2018; Mortier et al., 2018; Miellot et al., 2005; Middendorp and Nieuwenhuis, 2009; Jacques et al., 2010).

The microbiome's influence on signalling molecules is not limited to cytokines, it has been shown to produce a wide variety of hormones that enter systemic circulation and influence host physiology. Glucagon-like peptide-1 (GLP-1), GLP-2, peptide YY, ghrelin, and gastric inhibitory peptide (GIP) are all hormones that are produced by the microbiome (Nissen et al., 2014; Fukushima et al., 2005; Choi et al., 2013; Lafage Proust, 2017; Henriksen et al., 2009; Nuche-Berenguer et al., 2010). The impact of these hormones on bone health is still debated, however, human and animal studies have shown GLP-1, GLP-2, ghrelin, and GIP demonstrate anabolic and anti-catabolic effects on bone (Nissen et al., 2014; Fukushima et al., 2005; Choi et al., 2013; Lafage Proust, 2017; Henriksen et al., 2009; Nuche-Berenguer et al., 2010). While these hormones may not play a major role on their own, their imbalance may help tip the scales and create a favourable environment for increased bone turnover.

### 3.3 The influence of the microbiome on gut wall integrity and systemic circulation

In healthy states, the Gut-vascular barrier (GVB) and the gut-epithelial barrier function to ensure compartmentalization of gut contents and separate them from immune cells and stromal tissue located in the intestinal wall (Rizzo et al., 2018; Tajik et al., 2020; Ciccia et al., 2017; Martinez-Gonzalez et al., 1994; Tlaskalová-Hogenová et al., 2004; Tian et al., 2016; Morris et al., 2016). This barrier consists of adjacent epithelial cells connected by tight junctions, desmosomes, and adherens junctions, and serves to control and restrict antigen movement into systemic circulation. The microbiome exhibits a profound effect on the structural integrity of the intestinal walls, and dysbiosis disrupts expression of tight junction proteins such as claudin 4 and occludin, increasing both wall permeability and epithelial cell detachment from the basement membrane and apoptosis (Rizzo et al., 2018; Tajik et al., 2020; Ciccia et al., 2017; Martinez-Gonzalez et al., 1994; Tlaskalová-Hogenová et al., 2004; Tian et al., 2016; Morris et al., 2016).

This breakdown of gut structures allows for the translocation of bacterial products such as lipopolysaccharides (LPS) into systemic circulation and triggers a systemic immune response, contributing to the development and progression of autoimmunity (Rizzo et al., 2018; Ciccia et al., 2016). Reinforcing this finding, animal studies on RA models demonstrated that gut barrier function is disrupted prior to clinical presentation (Tajik et al., 2020). In these animals, zonulin was identified as a key molecule that increased intestinal permeability and allowed for translocation of gut contents and activated immune cells to synovial tissue. Notably, this study also identified that targeting and inhibiting zonulin resulted in a diminished arthritic presentation and was associated with disease improvement (Tajik et al., 2020).

The aberrant cellular trafficking theory states that chronic inflammation in the gut results in recruitment of T-cells to the inflamed sites (Fantini, 2009; Salmi et al., 1997; Hindryckx et al., 2011). In autoimmune conditions, this is facilitated by an increase in the expression of adhesive ligands. To highlight this point, IBDs see this adhesive effect amplified 10-fold, and patients with SpA also have an increase in expression of these molecules that facilitate T-cell honing to sites of inflammation (Fantini, 2009; Salmi et al., 1997; Hindryckx et al., 2011). Once in their target location, these immune cells are activated and upregulate cytokines and chemokines that prime them to preferentially distribute to sites in the body that mimic the initial area of activation and antigen recognition (Jacques et al., 2010). Studies have now shown that patients with IBD have cells that readily locate to synovial surfaces - indicating an underlying structurally similar feature that allows for cross-reactivity and concurrent targeting of the bowels and the joints (Salmi and Jalkanen, 2001; Ley et al., 2016).

In addition to impaired immune trafficking, disrupted gut wall barriers allow for migration of bacteria to joint spaces and allow for colonization of the synovial fluid. This phenomenon is seen in reactive arthritis and in SpA, so it is plausible that the translocation and colonization of the synovium by microbes facilitates immune infiltration and autoimmunity in these conditions (Berthelot and Claudepierre, 2016; Kempsell et al., 2001).

### 3.4 The influence of the microbiome on bone and disc health

The role of the microbiome and musculoskeletal health has gained increasing attention over the past decade. While the role of metabolites such as SCFA in this relationship has already been discussed, the microbiome also influences the absorption of nutrients such as calcium and vitamin D, which are essential for bone health (Sjögren et al., 2012). Moreover, studies have identified specific bacterial strains that are associated with healthy bones. *Bacteroidaceae*, *Lachnospiraceae*, *Porphyromonadaceae*, *Rikenellaceae*, and *Ruminococcaceae* have been seen in increased quantities in diseased states, whereas the levels of *Prevotellaceae* and *Veillonellaceae* decrease (Pedersen and Maksymowych, 2019; Costello et al., 2015). While the true role these bacterial families play is still unknown, their fluctuations in diseased states point to a possible relationship between dysbiosis and onset of disease (Pedersen and Maksymowych, 2019; Costello et al., 2015).

A particularly noteworthy finding from a comparative metagenomic analysis by Rajasekaran et al., is that there exists a distinct microbiome within human intervertebral discs (IVD), and this microbiome is distinctly altered in healthy and diseased discs (Rajasekaran et al., 2020). Not only does this finding challenge the notion that IVDs are sterile environments, but the study also showed that healthy discs exhibited an abundance of protective commensal bacteria while diseased discs showed pathogenic bacteria. The study identified 58 bacteria that were common between the IVD and gut, and 29 that were common between the IVD and skin. Between the skin and IVDs, *Acinetobacter johnsonii, Pseudomonas stutzeri, Staphylococcus epidermidis,* and *Corynebacterium durum* were among the most common bacteria, while *Prevotella copri* and *Faecalibacterium prausnitzii* were common in both the IVDs and GI tract (Rajasekaran et al., 2020). Although AS and related conditions are the primary focus of this review, Rajasekaran's findings offer additional evidence of the microbiome's influence on the structural components of the spine and offer valuable parallels. Findings with IVDs help expand the understanding of how dysbiosis and subclinical infections influence bone and spine pathology (Rajasekaran et al., 2020).

While this review primarily focuses on ankylosing spondylitis and related arthropathies, insights from studies examining the microbiome's impact on intervertebral disc (IVD) health offer valuable parallels. IVD-focused findings help expand the understanding of how dysbiosis may influence various spinal tissues beyond the joints.

A recent human study by Aboushaala et al. investigated the gut microbiome in patients with lumbar degenerative spondylolisthesis (LDS), identifying significant microbial differences between LDS patients and non-LDS symptomatic controls (Aboushaala et al., 2024). Notably, the relative abundance of *Firmicutes* and *Bacteroidetes* showed an inverse relationship, with LDS patients exhibiting a higher *Firmicutes:Bacteroidetes* ratio (p = 0.0031) (Aboushaala et al., 2024). This association remained statistically significant even after adjusting for factors such as age, BMI, and sex (p = 0.037). Furthermore, at the genus level, LDS patients had higher levels of pro-inflammatory bacteria, including *Clostridium* CAG-352, *Ruminococcaceae* UCG-010, and *Dialister*, and lower levels of beneficial anti-inflammatory taxa, such as *Oscillospira*, *Adlercreutzia*, *Lactobacillus*, and *Tyzzerella* (Aboushaala et al., 2024). These shifts in microbe composition indicate that gut dysbiosis may contribute to disc degeneration and LDS via the triggering of local inflammation (Aboushaala et al., 2024). These findings Aboushaala et al. serve to further support the theory that spine structures can serve as sites for bacteria to seed and potentially pathologically influence.

### 3.5 Key points

The microbiome plays a major role in immune regulation, gut barrier integrity, cytokine signaling, and bone health. Table 1 offers a brief summary of key players in the relationship between the microbiome and spine health and autoimmunity. Dysbiosis disrupts these functions and can contribute to chronic inflammation and autoimmune disease. Microbial byproducts such as SCFAs and the impact of bacterial populations on cytokines like IL-17 and IL-23 influence systemic immunity and are thought to help drive conditions like SpA and AS. Additionally, microbial compositions and signals affect gut permeability, enabling bacterial translocation to distant tissues such as joints. New evidence in the literature links gut dysbiosis to spine pathology through both downstream immune-mediated pathways and direct alterations in local spine microbiomes. Together, these findings highlight the different ways in which the gut microbiome may contribute to spine-related disease and draw attention to a novel relationship that ultimately requires further elucidation and exploration.

**Table 1:** **Key microbiome and immune factors relevant to spinal health:** The table below summarizes components involved in gut-immune interactions and their proposed relevance to spondyloarthropathies.

Key microbiome and immune factors relevant to spinal health		
Factor	Role in Gut/Immune Health	Implication for Spine Health
Dysbiosis	Alters gut permeability, dysregulates cytokines	Increase systemic inflammation
SCFA	Anti-inflammatory, promotes gut barrier integrity	Decreased levels in diseased states, repletion associated with symptom improvement
IL-17/IL-23	Promote mucosal immunity and inflammation	Dysregulation seen in SpA/AS pathogenesis
HLA-B27	Associated with immune hyperactivity states	Genetic risk factor for AS/SpA and multiple autoimmune conditions
MAIT/iNKT cells	First-line responders to microbial antigens	Play a role in the development of IBD and SpA. Present in increased numbers in joint spaces

## 4 Results: Ankylosing spondylitis, spondyloarthropathies, and their relationship with the microbiome

Spondyloarthropathies represent conditions where the potential relationship between the human microbiome and musculoskeletal health becomes evident in a clinically significant manner. SpAs consist of a wide range of inflammatory arthropathies that have a shared common clinical presentation and underlying genetic predispositions. Common clinical manifestations are sacroiliitis, back pain, and stiffness; however, these conditions are also characterized by axial and peripheral joint involvement (Sen et al., 2023).

Classification of SpA is according to the MRI-based Assessment of Spondyloarthritis International Society (ASAS), who divide this condition into axial SpA (axSpA), psoriatic arthritis, peripheral arthritis, IBD-associated arthropathies, and reactive arthritis (ReA). Axial SpA can be further subdivided into non-radiographic sacroiliitis, or radiographic sacroiliitis.

While the pathogenesis of AS is not fully understood, there is a strong genetic predisposition with Human leukocyte antigen B27 (HLA-B27). Genetic susceptibility alone, however, does not provide sufficient answers to the pathogenesis of AS. A significant proportion of AS patients also have concurrent gut inflammation and go on to develop IBD. This observation indicates a connection between these two conditions, and an understanding of the role of the microbiome in gut and intestinal inflammation will serve to provide a holistic view of this condition and provide improved patient-centred therapeutic strategies.

### 4.1 Relationship with inflammatory bowel disease

Inflammatory Bowel Disease (IBD) refers to a group of chronic, relapsing and remitting gastrointestinal conditions characterized by immune-mediated inflammation of the gut. There are two primary subtypes: Crohn's disease (CD), which can affect any part of the gastrointestinal tract and often involves transmural inflammation, and ulcerative colitis (UC), which is typically limited to the colon and involves superficial mucosal inflammation. The underlying aetiology of IBD is believed to involve a mix of genetic predisposition, environmental factors, changes in gut bacteria, and immune system overactivity.

There is a strong connection between SpA and IBD within the literature, with SpA being one of the most commonly reported manifestations of IBD (Speca and Dubuquoy, 2017). Roughly half of all patients with AS have some extent of gut inflammation, and around 10 % of these patients progress to develop IBD (Speca and Dubuquoy, 2017; Kabeerdoss et al., 2016). Gut inflammation is seen more commonly in axial SpA as compared to peripheral disease, and, as discussed earlier, dysbiosis and inflammation facilitate the entry of bacterial antigens into the mucosa, submucosa, and systemic circulation (Kabeerdoss et al., 2016).

The clinical and immunological overlap between IBD and spondyloarthropathies makes IBD an important condition when looking at the gut-immune-spine axis. This shared pathophysiology suggests that microbiome-driven intestinal inflammation may extend to extraintestinal sites, including the axial skeleton.

#### 4.1.1 Shared genetic and immunological factors

Increased levels of IL-23 are commonly seen in patients with AS, with sources of IL-23 including TH17 cells, Paneth cells, and monocytes (Kabeerdoss et al., 2016). This increase is observed to be pronounced further in ileal biopsies of AS patients who have concurrent Crohn's disease, compared to patients without IBD, suggesting that IL-23 plays a role in exacerbating gut inflammation and worsening the overall clinical course (Kabeerdoss et al., 2016). Moreover, IL-23 receptor gene single-nucleotide polymorphisms (SNPs) are associated with an increased risk of developing AS. This genetic predisposition to being more reactive and sensitive to IL-23, combined with underlying bowel inflammation and IBD, may lead to a more severe clinical course for those already susceptible to AS, highlighting the interplay between gut health and SpA (Kabeerdoss et al., 2016).

Further complicating and adding to the clinical picture, in chronic inflammatory conditions, there is increased chemotaxis of T cells to areas of inflammation (Kabeerdoss et al., 2016). These T cells are recruited to these sites by adhesion ligands such as VLA-4, CD44, and LFA-1. Immune cells derived from the gut of patients with IBD were observed to have nearly a tenfold greater capacity to attach to these adhesive molecules (Kabeerdoss et al., 2016). Thus, the heightened expression and activation of IL-23 and its receptor, combined with increased T cell homing and adhesion, creates a feedback loop that exacerbates both gut and joint inflammation.

#### 4.1.2 Genetic link with HLA-B27

HLA-B27 is a major histocompatibility complex molecule, it plays a role in presenting antigens to the immune system and is strongly associated with several autoimmune diseases - notably AS and IBD. As discussed earlier, increased gut permeability plays a possible role in the pathophysiology of AS. Research suggests that individuals with the HLA-B27 gene have inherently more permeable gut walls, with rodent studies showing a nearly five times higher permeability as compared to controls (Asquith et al., 2017; Kerr et al., 1999). In addition to its impact on gut wall integrity, HLA-B27 is thought to prime the body to be in a pro-inflammatory state. In response to insults, there is a greatly exaggerated immune response (Asquith et al., 2017; Hollander et al., 1986). A potential cause of this effect was identified with the impact of the anti-inflammatory SCFA propionate in rodent models. Interestingly, propionate has been found to cause decreased intestinal inflammation (Asquith et al., 2017). However, levels of propionate in HLA-B27 rodents were not decreased when compared to normal healthy controls, leading to the theory that HLA-B27 disrupts the receptors for propionate, thereby blunting its anti-inflammatory effect (Asquith et al., 2017).

The relationship between SpA, gut inflammation, and IBD is gaining increasing attention with nearly 10 % of all AS patients going on to develop clinically significant IBD. Animal studies have shown that under sterile, germ-free conditions, HLA-B27 rodents did not develop any manifestation of AS or IBD. Notably, upon reintroduction of a commensal microbiome, both intestinal and GI disease processes returned (Asquith et al., 2017; Cardoneanu et al., 2021). This finding serves to further emphasize the importance and role that the gut microbiome serves in the disease process of both conditions.

#### 4.1.3 Improper immune activation

As mentioned earlier, gut dysbiosis is closely followed by a disruption of gut metabolites. Improper dendritic cell recognition of these metabolites and antigens - particularly through HLA-B27 is thought to initiate Axial Spondyloarthritis (Berthelot and Claudepierre, 2016; Krajewska-Włodarczyk et al., 2017). Ex vivo studies of dendritic cells draw a parallel to reactive arthritis and describe a pathway of migration where bacteria trapped within dendritic cells have the potential to travel from the gut to the axial skeleton and begin an inflammatory reaction (Berthelot and Claudepierre, 2016). These foreign pathogens then trigger the immune response and result in the pathologic activation of these HLA-B27 positive dendritic cells. These dendritic cells have then been observed to initiate a massive IL-17-dominated immune response and drive the development of AS and SpA (Utriainen et al., 2012; Glatigny et al., 2012; Slobodin et al., 2019).

#### 4.1.4 Molecular mimicry

The observation that dysbiosis and infection often precede SpA suggests that there may be a possible role of molecular mimicry, similar to the relationship between Streptococcus pyogenes and rheumatic heart disease (Gracey et al., 2019). HLA-B27+ CD8 cells may cross-react with enteric bacteria, targeting endogenous self-antigens (Speca and Dubuquoy, 2017; Schittenhelm et al., 2015; Hermann et al., 1993). *Klebsiella*, *Enterobacter*, *Salmonella*, and *Shigella* species have shown interactions indicating shared structures with endogenous self-antigens (De Vries et al., 1992; Fielder et al., 1995; Welsh et al., 1980). It is in this context that HLA-B27+ CD8 cells may react with enteric bacteria and subsequently begin targeting self-antigens in the joints and synovium (Speca and Dubuquoy, 2017; Schittenhelm et al., 2015; Hermann et al., 1993).

### 4.2 Key points

**Fig. 1:**
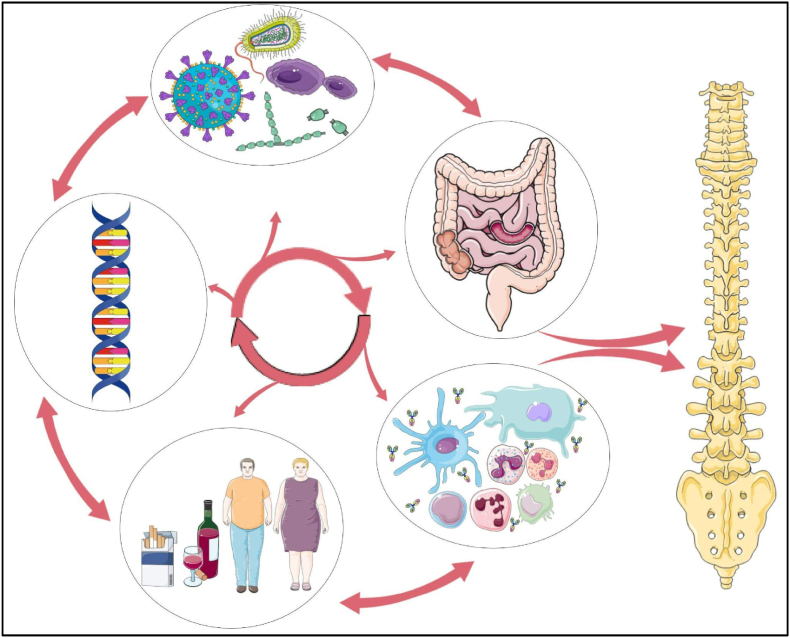
**The Gut-Spine Axis**: This figure represents the multifactorial relationship between the microbiome, immune system, host lifestyle, genetics, and gut health. These individual components are interconnected and influence one another. Disruption within this system can lead to immune dysregulation and altered gut function, both of which may contribute to the development and progression of spinal pathology.

**Fig. 2:**
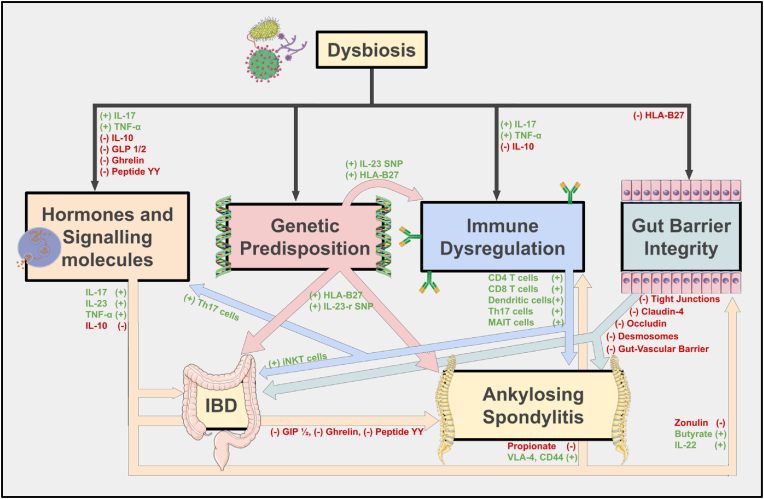
**Mechanisms Linking Dysbiosis to Gut and Spine Pathology:** This non-comprehensive diagram illustrates the pathways through which dysbiosis contributes to the development of autoimmune conditions such as Ankylosing Spondylitis (AS) and Inflammatory Bowel Disease (IBD). Dysbiosis disrupts signaling networks, including a reduction in protective short-chain fatty acids (SCFAs) like butyrate and propionate, and increases in pro-inflammatory cytokines such as IL-17, IL-23, and TNF-α. These changes create an environment that promotes systemic inflammation and impacts endocrine signaling, creating a self-sustaining inflammatory state. Compounding this, are genetic factors, such as HLA-B27 expression and IL-23 receptor single nucleotide polymorphisms (SNPs), which further increase immune reactivity to microbial antigens. Dysregulated immune cells, including TH17, MAIT, and iNKT cells, amplify inflammatory cascades. Dysbiosis also promotes gut barrier dysfunction, with loss of tight junctions, desmosomes, and epithelial integrity, allowing for microbial translocation into systemic circulation. This amplifies immune signaling and supports the honing of immune cells to sites such as joints and intervertebral discs, further linking gut health to spine pathology.

Within current literature, there exists a connection between the microbiome, inflammatory bowel disease, and spondyloarthropathies, particularly ankylosing spondylitis. The shared immunological features between gut and joint disease suggest a common pathophysiology influenced by underlying genetic predisposition, such as HLA-B27, and immune dysregulation. Evidence supports the role of immune cells derived from the gut and microbial antigens in initiating or worsening spinal inflammation. Mechanisms such as impaired immune activation and molecular mimicry arise as potential mechanisms that illustrate how gut disturbances may drive disease progression in susceptible individuals. As seen in Fig. 1, Fig. 2 below, dysbiosis and its downstream effects represent one facet of a multifactorial pathophysiology, and it is likely impairment in multiple domains that trigger disease progression. These findings support a growing theory of the existence of a gut-spine axis which uses the immune system as a mediator in the pathogenesis of AS and related conditions.

### 4.3 Future directions

Ongoing research aims to determine whether HLA-B27 plays a role in facilitating dysbiosis, or if it is dysbiosis that triggers the exaggerated immune response from HLA-B27. This relationship is complex and not fully understood, with evidence supporting both scenarios. There is the possibility of a two-hit hypothesis where a combination of genetic predisposition and environmental disruption through dysbiosis is what allows for pathogenesis of AS and associated arthritides. This model underscores the significance of both factors and highlights the need to further understand the underlying mechanism.

Future research should focus on establishing a solid foundational base of knowledge, as studies in the current literature often branch off from undefined starting points, taking diverse and unpredictable directions that do not contribute to or build a foundation of mutual understanding in the field. To avoid this, there is a crucial need for studies that establish a clear baseline of microbial knowledge. Longitudinal studies in human populations, using serial microbial sampling, would be particularly valuable. By identifying patterns in the microbiome over time, we could create a comprehensive understanding similar to the Framingham Heart Study for cardiovascular diseases. This approach would allow us to better identify the factors influencing the microbiome and how they may lead to alterations and dysbiosis.

In this foundation, it is essential to account for lifestyle factors that may shape the microbiome and modulate disease outcomes. Patient-specific factors such as diet, antibiotic use, and comorbidities like obesity and IBD can significantly alter the gut microbiome and may influence disease outcomes. Laying a solid base of microbiome knowledge would enable us to identify how these different lifestyle factors impact the microbiome and ultimately influence disease progression.

In addition to these foundational studies, more interventional trials in humans are needed. Exploring the effects of probiotics or fecal microbiota transplantation in AS patients, by replacing their microbiomes with those of genetically similar yet healthy individuals, could shed light on the role of the microbiome in disease severity and progression. The hypothesis being that replacing the dysbiotic microbiome with a healthier one may reduce disease severity and potentially alter disease progression, offering novel therapeutic strategies for AS.

Another potential avenue of future research involves further exploration of the *Firmicutes:Bacteroidetes* (F:B) ratio as a potential biomarker for spinal degeneration. In the study by Aboushaala et al., this ratio was significantly higher in LDS patients, even after adjusting for confounding factors. The F:B ratio is also reported to be four times higher in females, which may serve as an explanation for the gender disparity and higher prevalence of LDS in women (Aboushaala et al., 2024). Future studies should focus on elucidating the role of this ratio further and evaluate whether it may serve as a diagnostic biomarker and as a potential therapeutic target.

## 5 Discussion: Is the microbiome relevant?

The relevance of the microbiome to clinical practice is in its potential therapeutic and preventive medicine applications. Probiotics, prebiotics, and fecal microbiota transplantation are being explored as treatments to restore healthy microbiome balance and mitigate inflammatory responses. In SpA, preliminary trials with probiotics have shown some promise in reducing disease activity, although more extensive and controlled studies are needed to confirm these findings. For SpA, maintaining a healthy microbiome has the potential to reduce the risk of infections that could precipitate joint inflammation, providing a preventive strategy alongside traditional antimicrobial treatments (Cammarota et al., 2014; Charbonneau et al., 2016; Parvaneh et al., 2015).

SpA has been frequently associated with gut inflammation; patients often exhibit a disrupted microbiome and have an increase in proinflammatory bacteria and their metabolites. This dysbiosis is thought to trigger an immune response and start a cycle of inflammation that spreads to and exacerbates joint disease. The finding that genetically susceptible germ-free mice have demonstrated that the introduction of particular bacterial strains can exacerbate symptoms of SpA only serve to further emphasize the potential role that the microbiome may have in disease modulation.

A healthy and balanced microbiome can prevent the overgrowth of pathogenic bacteria through competitive exclusion. It also helps mature and regulate the immune system, thereby decreasing autoreactivity. Bacterial DNA and microbial infiltration observed in the joints of patients with SpA and AS provide evidence of bacterial invasion and infection within the joint spaces. This suggests that bacteria may play a direct or indirect role in the pathogenesis of these conditions.

Human pathology studies further support the significance of the microbiome in SpA and AS. Histological examinations often reveal gut inflammation and microbial infiltration in patients with SpA, while AS pathology typically shows evidence of bacterial infection within joint tissues. The presence of microbial DNA in affected tissues suggests a direct or indirect role of bacteria in the inflammatory processes underlying these diseases.

One notable challenge across studies looking at the gut microbiome that is worth being discussed is the overwhelming presence of conflicting or inconclusive findings. This variability is a result of heterogeneity in study populations, including differences in genetics, geography, lifestyle, and comorbidities. Dietary habits, medication use, particularly antibiotics and immunosuppressants, and iatrogenic interventions further complicate the individual microbial landscape. Moreover, inconsistencies in the methods used to characterize microbial populations can significantly affect results. Many studies study microbes only to the genus or family level, which can hide important differences at the species level. These finer taxonomic differences may carry distinct roles, with some strains producing metabolites that facilitate inflammation, while others don't.

However, the field is still in its infancy. Many findings have not been replicated in human models and are based primarily on ex vivo cell behaviour studies and rodent experiments. Current technology also limits the adequate measurement of the microbiome, often excluding critical components such as fungi, which are significant activators of the relevant cell lines. Demographic limitations also pose challenges, as most models connecting the microbiome to AS and SpA are based on Western populations, and findings have not been consistently reproduced in Eastern populations. Moreover, the personalized nature of the microbiome makes it difficult to compare gut makeup across different patients effectively.

## 6 Limitations

Research on the microbiome has gained increasing attention over the last 10 years. However, most research is yet to be applicable to clinical and human medicine.

The vast majority of the current understanding of immune cell interactions in relation to the microbiome is derived from preclinical studies, including animal models (particularly transgenic rodents), and ex vivo studies. While these methods provide essential insights to underlying mechanisms, their extrapolation to human physiology is challenging. The lack of human data makes it difficult to identify conclusive or generalizable results (Collin and Bigley, 2018; Clark et al., 2018). As a result, this review includes both preclinical and clinical studies due to the current limitations in existing literature. Thus, findings derived in regard to the microbiome should be approached with caution, especially when considering their clinical applications to humans.

The difficulty of studying immune cells important in AS and SpA pathogenesis is that these cells comprise less than 1 % of all cells in circulation, making it very challenging to isolate and identify their impact and underlying role in in-vivo studies. The current state of knowledge necessitates more human-focussed studies to better understand the true impact of these cells on human well-being, however, we may be technologically limited in the extent for which these cells can be isolated and studied.

Keeping with technological difficulties, the vast majority of microbiome studies focus primarily on bacterial compositions via 16s ribosomal RNA sequencing. This method overlooks other significant components of the microbiome such as viruses and fungi. This is particularly significant as these microbes play a potentially significant role in SpA pathogenesis as they are strong triggers for type 3 immune responses, especially the mycobiota as fungi can elicit a particularly robust type 3 immune response (Speca and Dubuquoy, 2017). Furthermore, current technology limits the extent to which the microbiome can be examined. Broad bacterial groups can be identified but specific strains and the exact makeup or each bacterial species is nearly impossible with current technology.

That being said, despite the fact that multiple type 3 immunity cell lines are linked to development of SpA, the exact roles of these cells in pathogenesis are still unknown. Data from studies aiming to identify key cytokines involved in type 3 immunity is inconclusive and the literature is far from any consensus on the role of the microbiome. For example, antagonizing IL-23 has no effect on axSpA but is seen to have some utility in treating psoriatic arthritis (Gracey et al., 2020). Blocking IL-17 is seen to be useful for management in all forms of SpA, but has been shown to serve little to no purpose in IBD (Gracey et al., 2020). These findings show that there might be multiple different mechanisms that are coming together to promote pathogenesis of SpA. In fact, further reinforcing this is the fact that as little as 30 % up to almost 60 % of patients on current therapies for SpA may not receive any benefit from treatment. This finding suggests that effective therapies act on the pathogenic process indirectly, and the true driving process is still unknown; with the observed link between SpA and type 3 immunity being coincidental. It is possible that type 3 immunity purely disrupts the gut wall and facilitates dysbiosis without affecting joint or bone health, suggesting that the same immune mechanism promotes both gut and joint inflammation, independent of each other, with gut inflammation preceding joint symptoms. This chronological order may be due to the soft tissue of the intestines being more malleable to the high inflammatory and microbial load, as compared to the stronger and slower reaction that mature mineralized bone and joint tissue have (Gracey et al., 2020).

The effects of the microbiome on the immune system are often mediated by metabolites rather than direct microorganism-host interactions. Currently, there is limited evidence of specific changes in the composition of the microbiome to SpA development. Certain bacteria, such as *Klebsiella pneumoniae* and *Bacteroides vulgatus* have been shown to play a hand in disease progression, but these results have been inconsistent and may instead reflect errors in study design or patient sample differences. Research should shift towards these metabolites to provide an understanding of how they influence disease progression and identify therapeutic targets (Costello et al., 2015). The focus of research should also be directed to broader changes in the overall microbiome and identify changes in immune and metabolic responses on a macro level instead of specific bacterial compositions.

The development of SpA without any form of bowel inflammation or dysbiosis is also a significant cause for concern regarding the role of the microbiome and the relationship between the gut and joints. A substantial proportion of SpA patients lack detectable levels of inflammation in the gut. Additionally, the role of genetics that is observed in Western European and North American populations has not been reproduced in Asian populations. Compared to their Western counterparts, East Asian populations not only have a lower prevalence of IBD but also a much lower prevalence of comorbid SpA and AS with IBD, with a less than 0.5 % prevalence of both diseases occurring concurrently in Chinese and Taiwanese patients as opposed to a rate hovering around 10 % for European patients (Gracey et al., 2020; Wang et al., 2017). This massive discrepancy challenges the current knowledge and models linking gut changes to SpA.

In summary, the literature on the microbiome and its role in SpA is fraught with methodological challenges and inconsistencies. Further research focusing on human models, broader microbiota components, and bioactive metabolites is crucial for advancing the understanding of the interplay between the gut and SpA. Study of the microbiome is complicated by its highly personalized nature. There is no standardized microbiome, and significant variation exists even among people from the same region with similar lifestyles and diets, making it difficult to draw cohesive and generalizable conclusions between results and studies. This personalization underscores the need for altered approaches in microbiome research and highlights it as the ultimate application of personalized medicine.

## 7 Conclusion

In conclusion, the microbiome is undeniably relevant to the clinical understanding and management of SpA and AS. Its influence on immune responses and potential as a therapeutic target underscores the need for continued research in this area. The burden of chronic disease is undoubtedly a growing challenge that needs to be addressed and by advancing knowledge of microbiome-pathogen interactions and their impact on human pathology, more effective strategies for diagnosis, treatment, and prevention of these debilitating conditions can be developed.

## Author contributions

All authors contributed equally to the conception and design of the study, interpretation of the available literature, and the drafting and revising of the manuscript. All authors have approved the final article for submission.

## Statements and declarations

None.

## Ethical considerations and informed consent

Not applicable.

## Source of funding

None.

## Declaration of competing interest

The authors declare that they have no known competing financial interests or personal relationships that could have appeared to influence the work reported in this paper.
